# Severe Hyperkalemia With Cardiac Conduction Abnormalities in a 92-Year-Old Woman: First Reported Case in Illinois, United States

**DOI:** 10.7759/cureus.95662

**Published:** 2025-10-29

**Authors:** Victor O Adedara, Samer Kholoki, Keenan Kassar, Chike C Amaechi, John Okei, Olayinka A Ojo, Oanthata Modise, Bryde E Ashuarrah

**Affiliations:** 1 Medicine, St. George's University School of Medicine, West Indies, GRD; 2 Internal Medicine, UChicago Medicine AdventHealth, La Grange, USA; 3 Medicine, Dominican University, River Forest, USA; 4 Medicine, Washington University of Health and Science, San Pedro, BLZ; 5 Medicine, Caribbean Medical University, Williamstead, CUW; 6 Medicine, All Saints University School of Medicine, Roseau, DMA

**Keywords:** cardiac arrythmia, cardiology research, hemodialysis, hemodialysis patients elderly, internal medicine (general medicine), severe hyperkalemia

## Abstract

Hyperkalemia, defined as a serum potassium level greater than 5.0 mEq/L, disrupts electrolyte balance and may cause neuromuscular symptoms, including palpitations, fatigue, or weakness. Severe cases exceeding 6.5 mEq/L can lead to life-threatening arrhythmias and cardiac arrest if left untreated. We present a 92-year-old female with stage 4 chronic kidney disease, diastolic heart failure, and a chronic right leg ulcer who arrived at the emergency department with lethargy and intermittent confusion following a fall. Laboratory results revealed a potassium level of 9.7 mEq/L, an estimated creatinine clearance of 9.4 mL/minute, a renal function index of 10.5, a bicarbonate level of 10.9 mEq/L, and a pH of 7.19. ECG demonstrated sinus bradycardia at 40 bpm, low-amplitude P waves, mild PR prolongation, and tall, narrow, symmetric T waves, most prominent in V2-V3 and multiple limb leads. The patient underwent emergent hemodialysis, which normalized her potassium level to 4.3 mEq/L within 24 hours. A repeat ECG showed resolution of abnormalities and restoration of a sinus rhythm at 95 bpm. This case highlights the life-saving importance of rapid ECG recognition, immediate laboratory confirmation, and prompt initiation of therapy in the management of severe hyperkalemia.

## Introduction

Severe hyperkalemia is a life-threatening electrolyte imbalance that occurs when serum potassium levels exceed 6.5 mEq/L. It can cause serious complications such as cardiac arrhythmias, muscle weakness, and, if untreated, cardiac arrest [1]. Clinical manifestations include muscle weakness, flaccid paralysis, and cardiac conduction abnormalities such as peaked T waves, widened QRS complexes, and ventricular arrhythmias. The underlying pathophysiology involves impaired renal excretion, reduced distal sodium delivery, aldosterone deficiency or resistance, and transcellular potassium shifts due to acidosis or tissue breakdown [2]. Hyperkalemia is a common and serious complication of chronic kidney disease (CKD), requiring close monitoring and prompt management to prevent adverse outcomes [3].

Standard treatments include intravenous calcium gluconate, insulin with glucose, and emergent dialysis. However, survival in extreme cases such as the present case with a potassium level of 9.7 mEq/L is uncommon [4]. Severe hyperkalemia often results from CKD or from medications that impair renal potassium excretion, including angiotensin-converting enzyme (ACE) inhibitors, angiotensin II receptor blockers (ARBs), mineralocorticoid receptor antagonists (MRAs) such as spironolactone and eplerenone, potassium-sparing diuretics, nonsteroidal anti-inflammatory drugs (NSAIDs), and potassium supplements. It may also develop secondary to acute illnesses that disrupt electrolyte balance [5]. While mild hyperkalemia is relatively common, extreme elevations are rare and carry a high risk of fatal cardiac events. Reporting survival in such cases reinforces the importance of early recognition and rapid intervention.

In the general population, the annual incidence of hyperkalemia (serum potassium ≥ 5.5 mmol/L) is approximately 0.96 per 100 person-years, affecting 2-2.3% of adults with at least one annual blood test [6]. Prevalence increases significantly in CKD: among adults with CKD stage G3, hyperkalemia (serum potassium > 5.0 mmol/L) occurs in 4.5-8.8%, rising to 23.7-34.4% in stage G5, with higher rates in patients with diabetes, according to the Kidney Disease: Improving Global Outcomes (KDIGO) guidelines [2]. Hyperkalemia is associated with increased risks of all-cause mortality, cardiac arrhythmias, and progression to kidney failure, with adjusted hazard ratios for mortality ranging from 1.5 to 2.3 depending on baseline kidney function [6].

To our knowledge, no previous case reports have documented survival at this degree of hyperkalemia (9.7 mEq/L) in the state of Illinois, United States. Although survival has been reported elsewhere at similar or higher levels, this appears to be the first documented case from Illinois. We present the case of a 92-year-old woman who survived profound hyperkalemia through prompt diagnosis and immediate initiation of hemodialysis.

## Case presentation

A 92-year-old woman, who lived independently, was brought to the emergency department by her son after a fall at home. She reported feeling lightheaded before losing her balance and striking the back of her head. She denied loss of consciousness or seizure activity but complained of right leg pain. Her son noted several days of progressive decline, including multiple episodes of diarrhea, poor oral intake, generalized weakness, and increasing confusion.

Her medical history was significant for stage 4 CKD, diastolic heart failure with peripheral edema, and a chronic right leg ulcer complicated by prior cellulitis. Her home medications included allopurinol, aspirin, atorvastatin, levothyroxine, losartan, potassium chloride, spironolactone, tiotropium, and torsemide. She had been taking both a loop diuretic and a potassium-sparing agent, along with oral potassium supplements, as part of her long-term regimen for severe lower extremity edema and diastolic heart failure. She was not on anticoagulants and had no recent exposure to nephrotoxic agents. There was no history of fever, chest pain, cough, abdominal pain, dysuria, or gastrointestinal bleeding.

On arrival, she was lethargic and intermittently confused. Vital signs revealed sinus bradycardia at 40 beats per minute, blood pressure of 110/68 mmHg, and respiratory rate of 18 breaths per minute. Physical examination demonstrated bilateral pitting edema up to the knees and a chronic right leg ulcer without erythema or discharge. Cardiac and pulmonary examinations were unremarkable, and the abdomen was soft and non-tender.

Neurological evaluation showed an altered mental status characterized by lethargy, intermittent confusion, and disorientation to time and place, without focal neurological deficits. These findings were consistent with metabolic encephalopathy, likely secondary to severe hyperkalemia and underlying renal dysfunction. The patient was arousable to verbal stimuli but exhibited slowed responsiveness and impaired short-term recall, which improved following correction of the electrolyte imbalance.

Investigations

**Figure 1:**
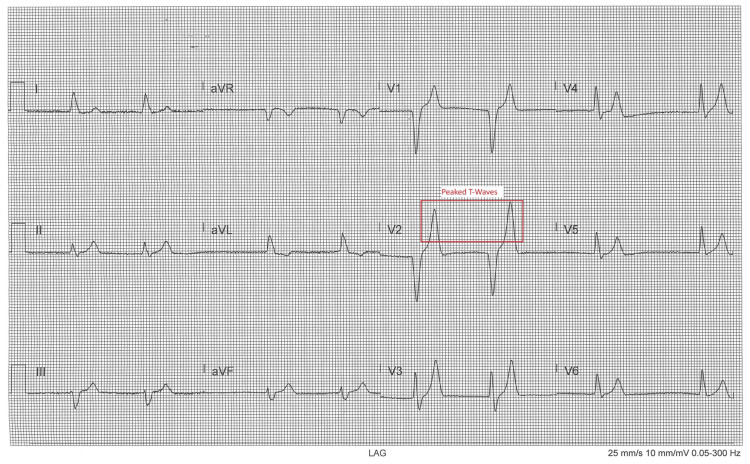
Electrocardiogram Before Hemodialysis Demonstrating Tall, Symmetric T Waves Consistent With Hyperkalemia

Electrocardiography revealed a sinus rhythm at approximately 40 beats per minute, characterized by low-amplitude P waves, most visible in leads II and V5-V6. The PR interval was mildly prolonged, the QRS complexes were widened, and there were marked repolarization abnormalities. These included tall, narrow-based, symmetric T waves that were disproportionately large relative to the QRS complex, most prominent in leads V2-V3 and evident across multiple limb leads. These findings were consistent with early to moderate hyperkalemia (Figure 1).

Initial laboratory testing demonstrated a markedly elevated serum potassium level. Repeat analysis confirmed a critically high concentration of 9.7 mEq/L. Renal function tests indicated acute kidney injury on top of CKD, and arterial blood gases showed metabolic acidosis.

Management and outcome

**Figure 2:**
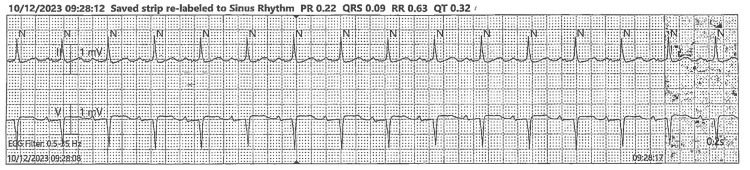
Post-hemodialysis ECG Demonstrating Regular Sinus Rhythm With Normal Depolarization and Repolarization

**Figure 3:**
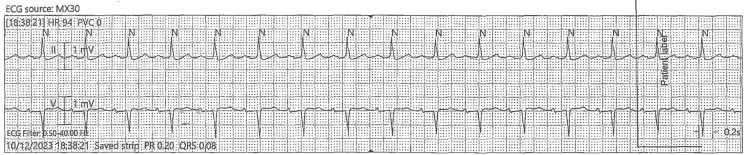
Post-hemodialysis ECG Showing Restoration of Regular Sinus Rhythm

Immediate activation of the hyperkalemia protocol was undertaken, and the patient was admitted to the intensive care unit for urgent intervention. She underwent emergent hemodialysis within hours of arrival. By the following day, her potassium level had normalized to 4.3 mEq/L. A repeat ECG at that time showed resolution of the hyperkalemia-associated changes, with restoration of a regular sinus rhythm at approximately 95 beats per minute. Computer measurements revealed a PR interval of 0.22 seconds, a QRS duration of 0.09 seconds, and a QTc of ~0.40 seconds, all within normal limits. The only persistent abnormality was mild PR prolongation, consistent with her baseline conduction pattern (Figures 2-3).

Throughout her hospital stay, the patient remained hemodynamically stable without progression to malignant arrhythmias. After stabilization and normalization of her potassium, she was safely transferred from the intensive care unit to the medical ward for ongoing monitoring of renal function and recovery. The summary of laboratory and ECG findings is shown in Table 1.

**Table 1:** Summary of Laboratory and ECG Findings

Parameter	Before Hemodialysis	After Hemodialysis
Serum Potassium (mEq/L)	9.7 (critically elevated)	4.3 (normalized)
Heart Rhythm	Sinus rhythm 40 bpm	Sinus rhythm ~95 bpm
P Waves	Low amplitude (II, V5-V6)	Normal amplitude
PR Interval	Mildly prolonged	0.22 seconds (mild prolongation, baseline)
QRS Duration	Widened	0.09 seconds (normal)
T Waves	Tall, narrow-based, symmetric (V2-V3)	Resolved, no hyperacute features
QTc Interval	Not prolonged	0.40 seconds (within normal limits)

This case highlights the critical importance of promptly recognizing and treating severe hyperkalemia, a condition that primarily threatens the cardiac conduction system and can rapidly progress to fatal arrhythmias or cardiac arrest. Serum potassium levels exceeding 9 mEq/L are typically associated with impending cardiac arrest and carry an exceptionally high mortality risk if not treated emergently. In this patient, immediate repeat confirmation and prompt initiation of hemodialysis were crucial for survival. Although pseudohyperkalemia must always be ruled out to avoid unnecessary intervention, any delay in therapy can be catastrophic. The presence of early ECG changes and concurrent metabolic acidosis further strengthens the need for aggressive and timely management to prevent irreversible cardiac complications.

Clinical implication

Because hyperkalemia with ECG changes is potentially unstable, treat according to protocol and monitor potassium/ECG levels serially. The ECGs do not show a malignant pattern at the captured moments, but the 12-lead confirms a potassium effect on repolarization, justifying calcium for membrane stabilization, plus temporizing shift/elimination measures as indicated. Continuous telemetry and repeat 12-lead after therapy to confirm resolution of the peaked T waves.

## Discussion

This case demonstrates the successful management of severe hyperkalemia (9.7 mEq/L) secondary to CKD with its definitive therapy in an elderly patient. The life-threatening cardiac manifestations seen on ECG, including widened QRS, tall T waves, and low-amplitude P waves, carried a significant risk for rapid multi-organ decline. These findings were reversed by emergent hemodialysis, which promptly restored cardiac function. Subsequent laboratory values showed a downward trend and stabilization of electrolyte abnormalities (Table 2).

**Table 2:** Serial Laboratory Values Demonstrating Electrolyte Abnormalities and Renal Function Trend On admission (October 11, 2023), the patient’s potassium was critically elevated at 9.7 mmol/L, consistent with severe, life-threatening hyperkalemia. After emergent hemodialysis, levels rapidly decreased to 4.1 mmol/L (October 12, 2023) and remained within the near-normal range during subsequent checks (4.9-5.0 mmol/L from October 13 to 17, 2023). A later outpatient value (July 8, 2024) showed a potassium level of 4.9 mmol/L, indicating long-term stability despite ongoing renal dysfunction. AGAP, anion gap; Alk Phos, alkaline phosphatase; ALT_SGPT, alanine aminotransferase (serum glutamic pyruvic transaminase); AST, aspartate aminotransferase; Abs, absolute; Bili, bilirubin; BUN, blood urea nitrogen; Est CrCL_CG, estimated creatinine clearance (Cockcroft-Gault); GFR CKD-EPI, glomerular filtration rate (Chronic Kidney Disease Epidemiology Collaboration); Hct, hematocrit; Hgb, hemoglobin; MCH, mean corpuscular hemoglobin; MCHC, mean corpuscular hemoglobin concentration; MCV, mean corpuscular volume; NRBC, nucleated red blood cell; Phos, phosphorus; RDW, red cell distribution width; RBC, red blood cell; WBC, white blood cell

	10/11/2023	10/12/2023	10/13/2023	10/17/2023	07/08/2024
WBC (×10^3^/µL)	6.6	4.28	3.88	5.20	-
RBC (×10^6^/µL)	3.44	2.69	2.90	3.20	-
Hgb (g/dL)	10.6	8.2	8.9	9.7	-
Hct %	35.3%	25.8%	28.5%	32.1%	-
Platelet (×10^3^/µL)	181	119	113	119	-
MCV (fL)	102.6	95.9	98.3	100.3	-
MCH (pg)	30.8	30.5	30.7	30.3	-
MCHC (g/dL)	30	31.8	31.2	30.2	-
RDW %	16.8%	15.8%	15.6%	14.5%	-
Neutrophils %	83%	81%	65%	72%	-
Lymphocytes %	10%	9%	20%	14%	-
Monocytes %	6%	9%	14%	11%	-
Eosinophils %	0%	0%	1%	2%	-
Basophils %	0%	0%	0%	0%	-
NRBC Auto %	0.5%	0.5%	0.0%	0.0%	-
Abs NRBCs (×10^3^/µL)	0.03	0.02	0.00	0.00	-
Neutrophils Abs (×10^3^/µL)	5.48	3.47	2.51	3.77	-
Lymphocytes Abs (×10^3^/µL)	0.63	0.39	0.77	0.74	-
Monocytes Abs (×10^3^/µL)	0.43	0.39	0.54	0.55	-
Eosinophils Abs (×10^3^/µL)	0.02	0.01	0.04	0.11	-
Basophils Abs (×10^3^/µL)	0.01	0.00	0.00	0.01	-
Sodium (mEq/L)	133	139	139	137	-
Potassium (mmol/L)	9.7	4.1	4.9	5.0	4.9
Chloride (mEq/L)	112	98	100	98	-
CO2 (mEq/L)	10	33	31	32	-
AGAP (mmol/L)	11	8.0	8.0	7.0	-
Glucose (mg/dL)	85	110	72	79	-
BUN (mg/dL)	149	36	36	52	103.9
Creatinine (mg/dL)	3.9	1.51	2.23	2.71	4.15
Calcium (mg/dL)	10.4	8.0	7.9	8.6	-
Est CrCL_CG (mL/minute)	9.4	24.4	16.5	13.6	-
GFR CKD-EPI (mL/minute/1.73 m^2^)	10.5	32.2	20.1	16.1	-
Albumin Level (g/dL)	3.8	2.8	2.8	2.6	-
Total Protein (g/dL)	7.4	5.6	5.6	5.5	-
Phos (mg/dL)	-	2.1	4.6	4.0	-
Alk Phos (Units/L)	140	96	119	112	-
AST (Units/L)	31	24	31	22	-
ALT_SGPT (Units/L)	23	16	18	12	-
Bili Total (mg/dL)	0.3	0.5	0.4	0.4	-

This highlights the effectiveness of hemodialysis in treating severe hyperkalemia and underscores the importance of continuous ECG and laboratory monitoring for timely intervention.

ECG serves as a useful tool for early risk stratification in hyperkalemia, with QRS prolongation being the most predictive of short-term cardiac events [7]. However, Mahana et al. describe a case of hyperkalemia with atypical ECG findings that challenge conventional diagnostic expectations [8]. Laboratory confirmation remains essential, and prompt correction is necessary, as medical stabilization may prove to be refractory. The marked improvement in laboratory values and ECG morphology within 24 hours of initiating dialysis in our case further supports the efficacy of this modality in life-threatening scenarios.

We suspected that the significant rise in serum potassium was related to the patient’s diuretic regimen, particularly the use of spironolactone as part of her daily medications without consistent monitoring. This sheds light on the importance of regular follow-up with a primary care provider, especially for patients with renal impairment, to prevent unchecked electrolyte disturbances and mitigate life-threatening complications. The combination of CKD and MRA therapy (spironolactone) is a well-established cause of refractory hyperkalemia in older adults [9].

Severe hyperkalemia arises from complex mechanisms with predominantly adverse outcomes. An et al. found that in-hospital mortality risk is highest in patients with infections, malignancy, and acute organ failure [2]. Our case reinforces current guidelines and contextualizes the treatment modalities that enabled rapid correction, prevented arrhythmia, and ultimately averted cardiac arrest. Nonetheless, it has several limitations. As a single patient observation, its generalizability is limited. The patient’s advanced age, comorbidities, and medication history may also limit the broader application.

Potassium levels should be checked at intervals tailored to each patient, taking into account their CKD stage, other health conditions, and current medications [10,11]. For those with advanced CKD, closer monitoring, such as weekly to monthly, may be needed until potassium levels stabilize [10,11].

This case highlights that even with a critically elevated potassium level of 9.7 mmol/L, survival is possible due to adaptive physiologic mechanisms, particularly in patients with CKD or advanced age. Literature suggests that individuals with advanced CKD gradually develop tolerance to sustained hyperkalemia, which may blunt the expected cardiotoxic effects, such as malignant arrhythmias or sudden cardiac death [12-14]. These adaptations include enhanced extrarenal clearance of potassium through the gastrointestinal tract, increased intracellular uptake mediated by insulin and catecholamines, and modifications in cardiac excitability that reduce susceptibility to arrhythmogenic triggers [12-14].

Further research should address these limitations, as well as the roles of time sensitivity, genetic biomarkers, and comorbidities that predispose patients to life-threatening hyperkalemia and arrhythmias. Even severe hyperkalemia with significant ECG abnormalities can be successfully reversed with prompt recognition and aggressive management protocols, particularly through emergent hemodialysis. Clinicians should maintain a low threshold for dialysis consultation when faced with severe hyperkalemia and cardiac manifestations, as delays in treatment may prove fatal.

## Conclusions

This case highlights the crucial role of timely diagnosis and prompt intervention in managing severe hyperkalemia. The patient’s recovery illustrates how heightened clinical vigilance, expedited laboratory testing, and prompt initiation of therapy can significantly reduce morbidity and mortality. Regular follow-up with a primary care provider is equally important, particularly for patients with renal impairment who are on combinations of medications such as diuretics and spironolactone that can predispose them to hyperkalemia. Remarkably, as of 2025, two years after the initial presentation, the patient remains alive, doing well, and has been successfully managed. This long-term outcome underscores the resilience of elderly patients even in the context of advanced CKD, and highlights the value of sustained monitoring and individualized care. Raising awareness of these principles may lead to improved outcomes in future cases.
